# Fourth-Party Evaluation of Third-Party Pro-social Help and Punishment: An ERP Study

**DOI:** 10.3389/fpsyg.2018.00932

**Published:** 2018-06-12

**Authors:** Jianbiao Li, Shuaiqi Li, Pengcheng Wang, Xiaoli Liu, Chengkang Zhu, Xiaofei Niu, Guangrong Wang, Xile Yin

**Affiliations:** ^1^Reinhard Selten Laboratory, China Academy of Corporate Governance, Business School, Nankai University, Tianjin, China; ^2^Nankai University Binhai College, Tianjin, China; ^3^International Business School, Tianjin University of Finance and Economics, Tianjin, China; ^4^Neural Decision Science Laboratory, Weifang University, Weifang, China

**Keywords:** pro-social behaviors, fourth-party evaluation, feedback-related negativity, third-party help, third-party punishment

## Abstract

Pro-social behaviors have been adequately studied by neuroscientists. However, few neural studies have focused on the social evaluation of pro-social behaviors, and none has compared the neural correlates of different pro-social decision evaluations. By fourth-party evaluation of third-party punishment/help dictator game paradigm, we explored the third-party pro-social behaviors and derived feedback-related negativity (FRN) from the electroencephalogram. Different from previous event-related potentials (ERP) studies, we simultaneously focused on two different third-party pro-social behaviors, which were called third-party help and third-party punishment. For the first time, we compared the different neural processes of fourth-party evaluation on third-party help and punishment. Behavioral results showed that fourth-party bystanders appreciated the help behavior of the third party even more than the punishment behavior. ERP results revealed that fourth-party bystanders’ FRN amplitudes were modulated by the third-party behaviors. Under the assignment condition (70:30) with help/punishment magnitude 45 and (90:10) with magnitude 80, the third-party help elicited a larger FRN than third-party punishment; whereas under the condition (90:10) with help/punishment magnitude 45, the difference between FRN amplitudes disappeared. These results indicated that fourth-party bystanders ultimately agreed more with helpful third parties; however, after they witnessed the norm violation, they expected the third parties to punish the norm violators immediately. This phenomenon appears only when the third-party actors can achieve justice between norm violators and victims.

## Introduction

The evaluation of pro-social behaviors accurately reflects the ethical standards of a society. Behavioral and experimental economics have achieved some insights on the social evaluation of pro-social behaviors using the experimental paradigm, in which the fourth-party bystanders may evaluate third-party help or third-party punishment behaviors (Raihani and Bshary, 2015). Subsequently, they found that, on one hand, third parties who took punishment action on selfish dictators or helped victims were rewarded by bystanders more frequently than third parties who did not respond to a selfish dictator or a victim. On the other hand, third-party helpers were more likely to be rewarded than third-party punishers.

Neuroscience studies have not explored the neuronal mechanisms underlying such behavioral outcomes. Existing neuroscience studies have examined the motivations, brain processes, and even genetic factors of third parties who punished norm violators (Strobel et al., 2011; Qu et al., 2014) and helped the victims (Hu et al., 2015). These studies also examined the effects of situations and individual differences or individual heterogeneity on these brain processes (Knoch et al., 2010; Sun et al., 2015; Morese et al., 2016; Mothes et al., 2016). However, none have focused on the behavioral and neurophysiological foundations of how fourth-party bystanders, also called social publics, perceived and evaluated these third-party pro-social behaviors. The current study aims to classify the brain processes of fourth-party bystanders when evaluating third-party pro-social behaviors by assessing neuronal markers (electroencephalogram: EEG). It also investigates the neural differences between the evaluations of pro-social help and punishment.

In the situation of norm violation, pro-social behaviors are actions that are executed by third parties and driven by their other regarding preferences (Buchan et al., 2006). Third parties may be concerned with two potential justice targets when thinking about achieving justice and taking pro-social actions (Gromet and Darley, 2009). According to the targets of other-regarding, third-party pro-social behaviors can be divided into two kinds: helping victims when they demonstrate compensatory concerns or punishing norm violators when they demonstrate punitive concerns (Leliveld et al., 2012; Gummerum et al., 2016).

Psychological and behavioral studies have investigated the motivational structure of third-party behaviors using several empirical and ingenious experimental paradigms (Fehr and Gächter, 2002; Boyd et al., 2003; Fehr and Fischbacher, 2003, 2004; Leliveld et al., 2012). In these studies, the dominant motive of third-party punishment is to maintain the social norm and benefit all the members of our human society. Third-party pro-social behaviors are best accounted for by the hypothesis that people promote the welfare of others as an ultimate end and not by alternative hypotheses that treat these behaviors as instrumental toward ulterior benefits, such as future reciprocation or gaining social approval (Fehr and Fischbacher, 2004).

However, from an evolutionary perspective, reciprocity and reputation cannot be excluded from third-party behaviors (Raihani and Bshary, 2015). Numerous theorists have shown that pro-social help, which comprises actions that benefit others at one’s own expense, can be sustained if help behaviors are made visible and the helper will receive helping in return (Milinski et al., 2001; Seinen and Schram, 2006; Tomasello and Vaish, 2013). As for third-party punishment, punitive reputation may play a crucial role in motivating third parties to take punishment actions. Moreover, individuals cooperate because the threat of punishment makes it beneficial for them to do so (dos Santos et al., 2011, 2013). Punishment also plays the role of a signal that shows that the punisher cares about others, is trustworthy, and shows sympathy (Ye et al., 2011; Jordan and Rand, 2017). Punishment can lead to long-term benefits if it influences the punisher’s reputation, thereby making the punisher more likely to receive help in future interactions (Jordan et al., 2016).

Neuroscience studies recently started to investigate the brain processes of third parties involved in pro-social behaviors. The pro-social decision-making process is associated with activity in the large-scale nervous system, which includes multiple prefrontal, limbic, and subcortical regions (Sanfey et al., 2003; Wang et al., 2016b; Li et al., 2017). Strobel et al. (2011) showed that third-party punishment elicited stronger activation in the ventral striatum compared with that when no punishment is implemented. They also found that when punishment occurs, the activity of the left dorsal lateral prefrontal cortex is weaker than when no punishment occurs. Hu et al. (2015) revealed that both third-party help and punishment activates the bilateral striatum. They also found that third-party help and punishment involves two different networks; specifically, third-party help involves the bilateral striatum and the right lateral prefrontal cortex, and third-party punishment involves the bilateral striatum and the left lateral prefrontal cortex as well as ventral medial prefrontal cortex. Recently, David et al. (2017) further investigated the different neural mechanisms underlying third-party help and punishment. They revealed that the dorsal anterior cingulate cortex showed higher response during the help (vs. punishment) choice when the (un-)fairness of the proposer’s offer was considered by the participants (i.e., offender-focused).

Several studies have used event-related potential (ERP) technique on assessing the neural processes of third-party behaviors because the use of EEG provides high temporal resolution, which is useful for further investigation on the neural processes of punishment decisions especially over the time course (Mothes et al., 2016). Qu et al. (2014) examined the effect of unfairness degrees and punishment decisions on Ne/ERN amplitudes. They found that the Ne/ERN amplitudes were more negative for not punishment decisions than for punishment decisions. Sun et al. (2015) used a similar experimental paradigm and found a medial frontal negativity (MFN) effect, and this effect was modulated by unfairness levels. Mothes et al. (2016) suggested that the amplitudes of feedback-related negativity (FRN) were more pronounced when participants witnessed unfair offers. Hence, MFN (including ERN and FRN) amplitudes, which were related to the activation of anterior cingulate cortex (ACC) (Nieuwenhuis et al., 2004), were sensitive to fairness norm violations, and participants elicited larger MFN effects when they did not take punishment actions.

All these neuroscience studies focused on the motivations and brain processes of third-party pro-social punishers (Qu et al., 2014) and the effects of individual differences, such as altruistic tendency and empathy (Sun et al., 2015; Mothes et al., 2016). These studies, however, did not investigate the third-party pro-social help behaviors or the social evaluations of third-party pro-social behaviors. Loke et al. (2011) partly discussed the evaluation of pro-social help. They found that neural correlates of bystanders’ evaluation about pro-social helping behaviors exist. However, the authors mainly focused on the comparison between evaluations of assistance or not when someone obviously needed help or not. Their study did not investigate the differences between pro-social help and pro-social punishment, and their experimental paradigm was not a norm violation paradigm.

We aim to explore the brain processes of the fourth-party evaluation of third-party pro-social behaviors under the situation of norm violation. Specifically, we attempt to investigate the neural differences between the evaluations of pro-social help and punishment by answering the following question: Do bystanders always consider helping victims (or punishing norm violators) a better choice than punishing (or helping)?

### FRN and Forth-Party Expectation on Third-Party Pro-social Behaviors

To this end, we used the ERP technology and an adopted third-party punishment/help dictator game paradigm, in which a fourth-party evaluator is added. The high temporal resolution of ERP allowed us to catch the initial psychological processes of fourth-party bystanders after witnessing the third-party behaviors. In the ERP analyses, we focused on the FRN, which is referred to as a negative-going ERP peak between 200 and 350 ms (Miltner et al., 1997; Sun et al., 2015) at the front to central recording sites in the vicinity of ACC. The ACC is considered to be sensitive to detecting cognitive conflicts (Liu et al., 2004; Nieuwenhuis et al., 2004). Studies showed that modulation in ACC, as well as DLPFC and lPFC activities following fair and unfair offers of proposers, plays an important role in pro-social help or punishment decisions (Strobel et al., 2011; Hu et al., 2015; David et al., 2017).

Recent EEG studies examined the role of ACC-related FRN or ERN component in pro-social behavior scenarios (Qu et al., 2014; Sun et al., 2015; Mothes et al., 2016). They assumed that the FRN component is an indicator that reflects whether outcomes matched expectations (Oliveira et al., 2007; Sun et al., 2015; Mothes et al., 2016). When outcomes were unexpected, a larger FRN was elicited compared with those in the expected outcomes (Sun et al., 2015). Moreover, some studies demonstrated that FRN was elicited even when participants witnessed other individual’s behaviors (Yeung et al., 2005; Koban and Pourtois, 2014). Thus, FRN is a reliable indicator even in the perspective of fourth-party bystanders. We hypothesize that if the fourth party expect third-party actors to punish the norm violators, then third-party punishment will elicit a smaller FRN than third-party help, which goes against the expectation of the fourth party. Conversely, if the fourth party expect third-party help more, the help will elicit a smaller FRN than the punishment.

### FRN and Fourth-Party Evaluation Scores

The second point we are interested in is that whether the FRN amplitude characteristics of the fourth party following the third-party actions will predict fourth-party evaluation scores. Given that the ERP has high temporal resolution, studies mostly focus on the characteristic of EEG within 1 s or even 800 ms following the epochs. In such a limited time, few cognitive resources were used in the brain processes of individuals, and thus, they are cognitively constrained (Cappelletti et al., 2011). Therefore, in a dual-system perspective, the individual’s deliberative capacity was limited in a short time and their expected actions might be different from the situation when time is sufficient (Blechert et al., 2012). As emotion was considered to be a determining factor of the automatic processes, in a short time, individual’s expected behaviors were more possibly modulated by emotional reactions spontaneously (Qu et al., 2014; Yang et al., 2017). Bystanders would rapidly elicit empathic anger at witnessing injustice or harm to someone else, and the empathic anger is considered as a motivation underlying third-party punishment and the expectation of third-party punishment (Fehr and Gächter, 2002; Fehr and Fischbacher, 2004; Batson et al., 2007; Van Doorn et al., 2014; FeldmanHall et al., 2015). However, the evaluation of third-party help requires more cognitive resources (Loke et al., 2011; Erlandsson et al., 2014). Thus, the ERP characteristics, which were extracted in a time shorter than 1 s mostly reflected the brain processes involving third-party punishment evaluation compared with help evaluation. We expect that smaller FRN amplitudes following third-party actions may not always predict higher fourth-party evaluation scores.

We addressed the above issues in two studies. In Study 1, participants witnessed the third party punish the unfair dictator or help the victim receiver, when the third party can reach a fair between the dictator and the victim under somewhat unfair offer condition and cannot reach a fair under extremely unfair offer condition. In Study 2, participants can turn the unfair offer into a fair one under extremely unfair offer condition.

## Materials and Methods

### Study 1: Third Party Can Reach a Fair Under Somewhat Unfair Condition and Cannot Do That Under Extremely Unfair Condition

#### Participants

A total of 24 healthy volunteers from Nankai University participated in this study for monetary compensation. Three subjects were excluded due to technical problems and severe artifacts in the EEG data. The brain activities of 21 subjects (13 women, 8 men; mean age = 23.3 years; range = 21–25 years) were fully analyzed. All participants were right-handed and native Chinese speakers. They had normal or corrected-to-normal vision and had no history of psychiatric or neurological disorders. Written informed consent was obtained before we conducted the experiment. The study protocol was approved by the Ethics Committee of Business School of Nankai University.

We used E-Prime experimental program to present the game. Color bars were applied to present the assignments of the dictator and the final payoffs of the dictator and the receiver after the third party took actions. The horizontal viewing angle of each target picture was 3°, and the vertical viewing angle was 1.5°.

#### Stimuli and Task

We introduced fourth-party bystanders in a third-party punishment of dictator game to adopt a modified paradigm of this game (Raihani and Bshary, 2015; Mothes et al., 2016). In the experiment, the main unit of analysis was defined as a “trial,” where four persons were referred to as “dictator,” “receiver,” “third party,” and “bystander”. However, neutral terms (P1, P2, P3, and P4) were used in the experimental instructions. Participants were assigned the role of fourth-party bystander. In each trial, they first witnessed the decision of the dictator who distributed 100 Yuan between himself and the receiver. Then they witnessed the decisions of third parties that can turn the unfair offer into a fair one. Thus, third parties were given a starting endowment of 50 Yuan. Third parties were given the opportunity to adjust the initial distribution. They can pay 15 Yuan to reduce the dictator’s bonus by 45 (i.e., TPP) or to increase the receiver’s bonus by 45 (i.e., TPH).

Subsequently, the participants can rate the third-party actions using a five-point Likert scale. The score determined the magnitude that the participants agreed with the third-party actions. A score of “1” indicated that the participant strongly disagreed with the third-party’s decision, “5” indicated that the participant strongly agreed with the third-party’s decision, and “3” was a neutral score.

We presented two predetermined assignments (70:30) and (90:10). In the (70:30) situation, the TPH or TPP actions can achieve almost absolute fairness between the dictator and the receiver. On the one hand, if the third party punished the dictator, the payoffs of the dictator and the receiver were 25 and 30, respectively. On the other hand, if the third party helped the receiver, the payoffs of the dictator and the receiver were 70 and 75, respectively. In the (90:10) situation, third-party behaviors could hardly achieve fairness between the dictator and the receiver. The TPH action resulted in payoffs (90:55) and the TPP in (45:10). The 2 × 2 conditions were fulfilled to compare the ERP responses with the fourth-party evaluation of TPH and TPP, which realized or at least attempted to realize the fairness between the dictator and the receiver.

With each condition containing 40 trials, a total of 160 experimental trials were performed. We randomly interspersed 40 control trials between these 160 trials to prevent anticipation effect of the participants. In these control trials, the decision of the third party was neither to punish the dictator nor to help the receiver. We did not analyze the EEG of these control trials.

#### Procedures

**FIGURE 1:**
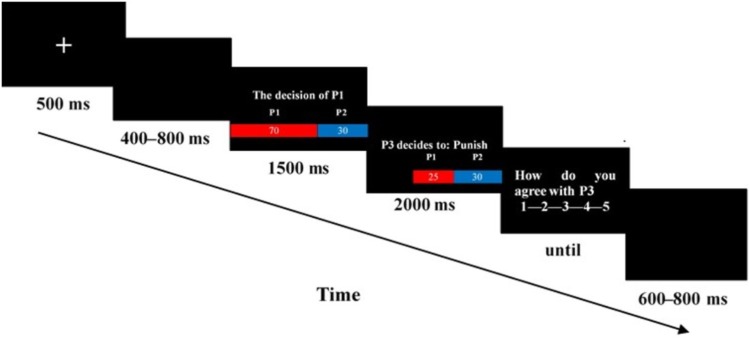
Time course of a single trial in Study 1. Each trial began with a 500-ms fixation point followed by the blank screen, which were randomized between 400 and 800 ms. A screen displaying the decision of P1 was shown for 1500 ms. Then, the stimulus presentation was shown for 2000 ms. Afterwards, an evaluation screen appeared until the participants responded. The intertrial interval was randomized to last between 600 and 800 ms.

Electroencephalogram recording was conducted in a small, sound-attenuated, and electrically shielded chamber. After EEG electrodes were attached, participants sat in a comfortable chair approximately 100 cm in front of a 23-inch computer monitor. Before the tasks began, all participants read the instructions carefully and were asked to take one or more 5-trial practice until the tasks were understood. **Figure 1** shows the time course of a single trial. Each trial began with the presentation of a single centrally located white fixation cross for 500 ms. Next, a blank screen was presented for 400–800 ms. Afterwards, the decision of the dictator, that is, to distribute 100 Yuan between himself and the receiver, was presented. Subsequently, decisions of the third party and the payoffs of the dictator and the receiver were presented at the center of the screen for 2000 ms. After the ERP, an evaluation display with five options (1, 2, 3, 4, and 5) was shown until the participants pressed the button of a five-key response pad.

The entire experiment comprised 160 test trials, 40 control trials, and 5 practice trials. Only the test trials were used for ERP analysis. Trials appeared in five blocks of 40 trials. Each block was separated by a break, the duration of which was determined by the participant. All 200 trials were performed within 15–25 min, during which these trials were randomly presented. E-Prime software was used to control the display of the stimuli and the acquisition of behavioral data (Version 2.0, Psychology Software Tools, Inc.).

#### EEG Acquisition

The EEG was recorded continuously with a 40-channel NuAmps DC amplifier (Compumedics Neuroscan, Inc., Charlotte, NC, United States). According to the International 10–20 System, 32 active Ag/AgCl electrodes were used. The impedances of all electrodes were kept below 10 kΩ. The reference electrode and the ground electrode were positioned at AFz. Electrodes below and above the left eye, as well as those located on the outer canthi of each eye, measured the bipolar vertical and horizontal electro-oculogram activities. Meanwhile, online EEG was digitized at a sampling rate of 1000 Hz using a 22-bit A/D converter.

Further offline processing was performed with Neuroscan Curry Software (Version 7.0.11, Compumedics Neuroscan, Inc., Charlotte, NC, United States). While offline, the reference of EGG signals was reset to the average of the left and right mastoids. Eye-blink artifacts were corrected, and the artifact rejection method excluded epochs with the EEG amplitude of any channel exceeding ±100 μV. The EEG data were band-pass filtered between 0.1 and 30 Hz. Subjects had no fewer than 40 artifact-free epochs in each condition, and the accepted epochs were baseline corrected. For each stimulus, we extracted 1000 ms epochs, with a 200-ms pre-stimulus period used as baseline.

#### EEG Analysis

The 1000-ms epochs were extracted in the markers “P3 decides to: Punish” and “P3 decides to: Help” starting at 200 ms before presentation of the third-party decisions. Mean amplitudes were then used for the FRN analysis. We found that maximum amplitudes of FRN were obtained at approximately 300 ms after participants witnessed third-party decisions over multiple frontal electrodes by visual inspection of grand averaged waveforms under TPH and TPP conditions. We then selected three electrodes in the midline area (Fz, FCz, and Cz) for statistical analysis (Mothes et al., 2016; Navarro-Cebrian et al., 2016). Previous studies suggested that maximum FRN amplitudes were often observed at mediofrontal electrodes, which corresponded to our observation (Gehring and Willoughby, 2002; Mothes et al., 2016). To further investigate the ERP characteristics, data from these three electrodes in a 270–330-ms time window were used.

For all analyses of variance (ANOVA), *p*-values were corrected using the Greenhouse–Geisser correction whenever the sphericity assumption has been violated. *p* < 0.05 was considered significant. Significant interaction was analyzed by the simple-effect model. Bonferroni correction was implemented to adjust for multiple comparisons. Statistics were analyzed with the IBM SPSS 19.0 software.

### Study 2: Third Party Can Reach a Fair Under Extremely Unfair Condition

#### Participants

A total of 19 healthy volunteers (10 women, 9 men; mean age = 22.8 years; range = 19–24 years) from Nankai University participated in Study 2. Their brain activities were all fully analyzed. In contrast to Study 1, numbers, not color bars, were applied to present the assignments of the dictator. The horizontal viewing angle of each target picture was 3°, and the vertical viewing angle was 1.5°.

#### Task and Procedure

The same game was used as in Study 1, except for the magnitude of TPH and TPP, which changed. In Study 2, third parties were given a starting endowment of 50 Yuan. They can pay 20 Yuan to reduce the dictator’s bonus by 80 (i.e., TPP) or to increase the receiver’s bonus by 80 (i.e., TPH). The payoffs of dictators can be cut down to 0 but can never be below 0. Thus, third-party actors can achieve fairness between the dictator and the receiver in the (90:10) situation. The TPH action resulted in payoffs (90:90) and the TPP in (10:10).

With each condition (TPH and TPP) containing 50 trials, a total of 100 experimental trials were performed. We randomly interspersed 40 control trials among these 100 experiment trials. In these control trials, allocations (50:50), (65:45), (70:30), (95:5), and (100:0) were included. All trials added up to 140.

**FIGURE 2:**
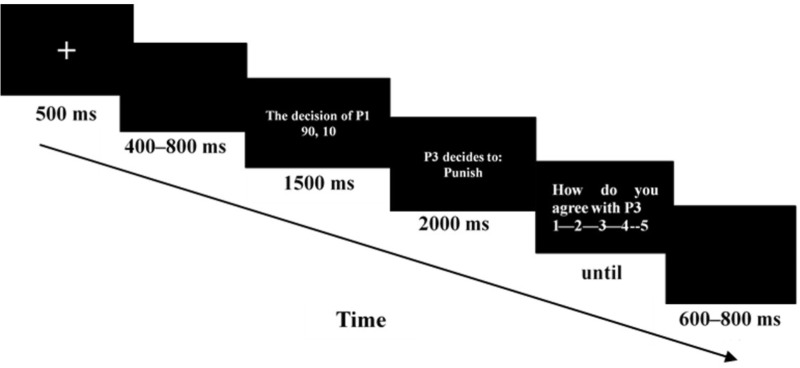
Time course of a single trial in Study 2. Each trial began with a 500-ms fixation point followed by blank screen, which were randomized between 400 and 800 ms. A screen displaying the decision of P1 was shown for 1500 ms. Then, the stimulus presentation was shown for 2000 ms. Afterwards, an evaluation screen appeared until the participants responded. The intertrial interval was randomized to last between 600 and 800 ms.

The procedure for each trial in Study 2 was the same as that in Study 1. The differences were that we replaced the color bars with numbers and removed the presence of final payoffs. **Figure 2** shows the time course of a single trial in Study 2.

The entire experiment comprised 100 test trials, 40 control trials, and 5 practice trials. Only the test trials were used for ERP analysis. Trials appeared in three blocks of 40 trials and one block of 20 trials. Each block was separated by a break, the duration of which was determined by the participant. All 140 trials were performed within 15–20 min, during which these trials were randomly presented.

#### EEG Acquisition and Analysis

The EEG acquisition and offline processing were the same as that in Study 1. The epoch selection was also similar. We found that the maximum amplitudes of FRN were obtained at approximately 300 ms after participants witnessed third-party decisions over multiple frontal electrodes by visual inspection of grand averaged waveforms under TPH and TPP conditions. We also selected three electrodes in the midline area (Fz, FCz, and Cz) for statistical analysis. To further investigate the ERP characteristics, data from these three electrodes in a 270–350-ms time window were used, which was different from the procedure in Study 1.

## Results

### Study 1: Achieving Fairness Under (70:30) and Not Achieving Fairness Under (90:10)

#### Behavior Results

For the assignment (70:30), 85.71% (18/21) of the fourth-party bystanders evaluated the TPH better than TPP, 14.29% (3/21) of the fourth party considered that TPH was nearly the same as TPP, and none of the bystanders preferred TPP. For the assignment (90:10), 66.67% (14/21) of the fourth-party bystanders rated the TPH higher, 4.76% (1/21) of the fourth party considered that TPH was nearly the same as TPP, and 28.57% (6/21) of the bystanders preferred TPP.

**FIGURE 3:**
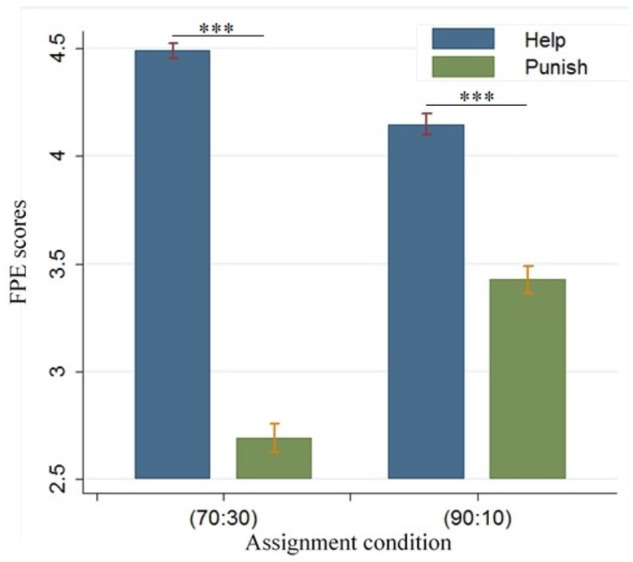
Behavioral results of fourth-party evaluation. Fourth-party evaluation scores are differentiated among (70:30, 90:10) × (TPH, TPP) conditions. “*” represents that p < 0.1, “**” represents that p < 0.05, and “***” represents that p < 0.01.

The fourth-party evaluation was performed using 2 × 2 repeated measures ANOVA with factors assignments (70:30, 90:10) and third-party behaviors (TPH vs. TPP). Significant effects [*F*(1,832) = 41.672, *p* < 0.001] and [*F*(1,832) = 736.341, *p* < 0.001] were yielded for factor condition (70:30, 90:10) and (TPP, TPH) (see **Figure 3**). A significant interaction effect occurred between first-party assignments and third-party behaviors [*F*(1,832) = 312.219, *p* < 0.001]. The fourth-party evaluation of TPH (mean = 4.49, *sd* = 0.725) was higher compared with the fourth-party evaluation of TPP (mean = 2.69, *sd* = 1.389) under the condition of (70:30) [*t*(832) = 39.534, *p* < 0.001]. We also found that the fourth-party evaluation of TPH (mean = 4.15, *sd* = 1.050) was higher than that of TPP (mean = 3.43, *sd* = 1.329) under the condition of (90:10) [*t*(839) = 10.301, *p* < 0.001].

We performed cluster regressions under condition (70:30) and (90:10) separately (see **Table 1**, A). In these regressions, we used the third-party behaviors as independent variables, the forth-party evaluation as dependent variables and participants as cluster indicators. We found the results were similar with those of ANOVA, the forth-party evaluation was more positive when third-party behavior was TPH compared to TPP under the condition of (70:30) (coef. = -1.809, *p* < 0.001), and the difference was also found under condition of (90:10) (coef. = -0.721, *p* = 0.073). The fourth-party bystanders agreed to the help behavior of the third party even more than punishment.

**Table 1:** Regression results of fourth-party evaluation.

Condition Variables	A		B
	(70:30)	(90:10)	(90:10)
		
	Forth-party evaluation		Forth-party evaluation
Third-party behaviors	-1.809***	-0.722*	-1.675***
	(0.255)	(0.382)	(0.313)
Constant	4.499***	4.148***	4.652***
	(0.135)	(0.197)	(0.0924)
Observations	1,656	1,680	1,883
*R*-squared	0.375	0.056	0.370

A is the cluster regression result of study 1. B is the cluster regression result of study 2. “Third-party behaviors” is a dumb variable in which “0” represents TPH and “1” represents TPH. “*” represents that p < 0.1, “**” represents that p < 0.05, and “***” represents that p < 0.01.

#### ERP Results

##### FRN: 270–330 ms

We assessed the ERPs evoked by TPH and TPP under the assignment conditions of (70:30) and (90:10). We submitted stimulus-induced activity in the FRN time range to 2 × 2 × 3 repeated measures ANOVA with the factors of first-party assignments (70:30, 90:10), third-party behaviors (TPH vs. TPP), and sites (Fz, FCz, and Cz). However, no significant differences were found between the (70:30) and (90:10) conditions [*p* > 0.05] and among electrodes [*p* > 0.05]. A significant difference was found between the TPH and TPP conditions [*F*(1,20) = 16.652, *p* = 0.001]. A significant interaction effect occurred between first-party assignments and third-party behaviors [*F*(1,20) = 7.794, *p* = 0.011]. We divided the data into two parts based on the first-party assignments and examined the difference between TPH and TPP.

##### FRN: (70:30)

**FIGURE 4:**
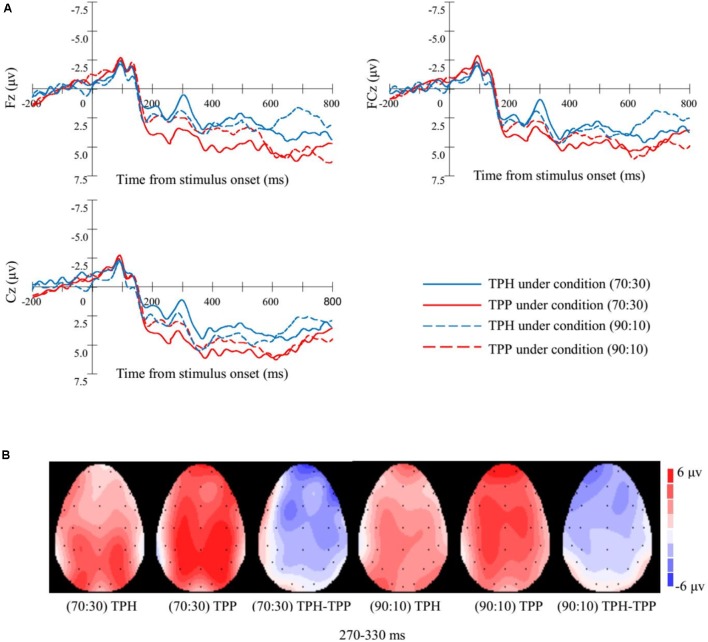
Event-related potentials (ERP) waveforms and topographic voltage maps of study 1. **(A)** Grand average ERP waveforms at selected electrodes of Study 1. ERP differentiated between TPP and TPH conditions: FRN between 270 and 330 ms at central sites (Fz, FCz, and Cz). **(B)** Topographic voltage maps of mean amplitude of Study 1. Voltage maps showing the scalp distributions with significant effects of (70:30, 90:10) × (TPH, TPP) obtained in the 270–330 ms epochs, and the difference between TPH and TPP (i.e., TPH-TPP).

Under the condition of (70:30), we conducted 2 × 3 repeated measures ANOVA with factors of third-party behaviors (TPH vs. TPP) and sites (Fz, FCz, and Cz). The result showed no significant effect of electrodes and no significant interaction effect between third-party behaviors and electrodes (all *p* > 0.05). Activity in the FRN time range was significantly more negative when the fourth-party bystanders witnessed TPH than when they witnessed TPP, as indicated by a main effect of third-party behaviors [*F*(1,20) = 36.571, *p* < 0.001]. Thus, the typical FRN of fourth-party bystanders was observed, and its topography is illustrated in **Figure 4**.

##### FRN: (90:10)

Under the condition of (90:10), we conducted 2 × 3 repeated measures ANOVA with factors of third-party behaviors (TPH vs. TPP) and sites (Fz, FCz, and Cz). The result showed no significant effect of electrodes and no significant interaction effect between third-party behaviors and electrodes (all *p* > 0.05). No significant difference between TPH and TPP was also observed [*p* > 0.05]. Statistical *post hoc* tests showed that the different waves were not significantly different from zero on all three electrodes (all *p* > 0.05).

### Study 2: Achieving Fairness Under (90:10)

#### Behavior Results

The fourth-party evaluation was analyzed using ANOVA with third-party behaviors (TPH vs. TPP) under allocation (90:10). Significant effects [*F*(1,1881) = 1107.06, *p* < 0.001] were obtained for factor condition (TPP and TPH). The fourth-party evaluation of TPH (mean = 4.651, *sd* = 0.679) was higher compared with the fourth-party evaluation of TPP (mean = 2.977, *sd* = 1.389) [*t*(1881) = 33.273, *p* < 0.001]. The fourth-party bystanders agreed to the help behavior of the third party even more than punishment. We also performed a cluster regression in which the third-party behaviors were independent variables, the forth-party evaluation were dependent variables and the identities of participants were cluster indicators (see **Table 1**, B). We found that the forth-party evaluation decreased when the third-party behaviors changed from TPH to TPP (coef. = -1.675, *p* < 0.001).

#### ERP Results

##### FRN: 270–350 ms

**FIGURE 5:**
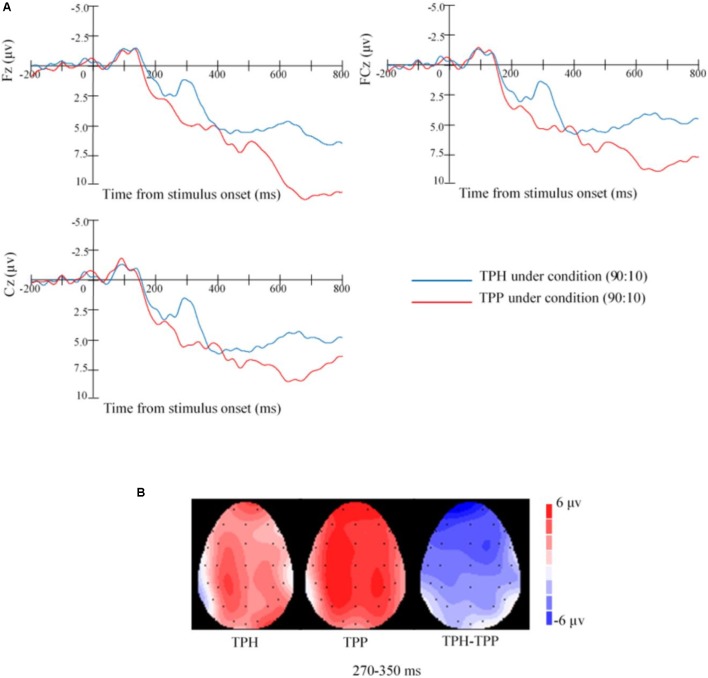
Event-related potentials waveforms and topographic voltage maps of study 2. **(A)** Grand average ERP waveforms at selected electrodes of Study 2. ERP waveforms that are differentiated between TPP and TPH conditions: FRN between 270 and 350 ms at central sites (Fz, FCz, and Cz). **(B)** Topographic voltage maps of mean amplitude of Study 2. Voltage maps showing the scalp distributions of significant effects of (TPH, TPP) obtained in the 270–350 ms epochs and the difference between TPH and TPP (i.e., TPH-TPP).

We assessed the ERPs evoked by TPH and TPP under the assignment conditions of (90:10) and the punishment or help magnitude 80 condition. We submitted stimulus-induced activity in the FRN time range to 2 × 3 repeated measures ANOVA with third-party behaviors (TPH vs. TPP) and sites (Fz, FCz, and Cz). A significant difference was found between the TPH and TPP conditions [*F*(1,18) = 26.134, *p* < 0.001]. However, no significant differences were found among electrodes, and no significant interaction effect occurred between third-party behaviors and electrodes (all *p* > 0.05). The typical FRN of fourth-party bystanders in Study 2 was observed and its topography is illustrated in **Figure 5**.

## Discussion

Interest in behavioral and neurophysiological research on pro-social behaviors has been growing in recent years. However, very few related studies focused on the issue of how social publics perceived and evaluated pro-social behaviors and the neural correlates. To understand the possible explanatory and modulatory factors of fourth-party evaluation of pro-social behaviors, our study examines the interplay between pro-social behavior types (i.e., third-party help and punishment), fourth-party evaluations, and the FRN component. Our study is the first to investigate the ERP correlates of social public evaluations on different kinds of pro-social behaviors.

### First Expectation: Agreement/Disagreement of Fourth-Party With the Third-Party Help/Punishment

In line with our first expectations, the behavioral data demonstrated that the fourth-party participants showed different feelings regarding third-party help and punishment. A comparison of the evaluation scores indicated that fourth parties agreed to third-party help more than punishment regardless of the first-party assignment decisions and the punishment or help magnitudes. The results corresponded to the concept of Raihani and Bshary (2015), who first discussed the fourth-party evaluation on third-party behaviors.

We examined the relation between the fourth-party evaluation, pro-social behavior types, and the FRN component associated with ACC-dependent responses toward unexpected outcomes (Hauser et al., 2014). The ERP data illustrated that a more negative FRN was exhibited by third-party help compared with punishment between 270 and 330 ms under assignment condition (70:30) with punishment/help magnitude of 45 and assignment condition (90:10) with punishment/help magnitude 80. Given that previous studies found that larger FRN amplitudes were observed for unexpected or surprise events (Oliveira et al., 2007; Sun et al., 2015; Mothes et al., 2016), we can deduce that third-party punishment is more likely to be expected by the fourth-party bystanders than third-party help.

Feedback-related negativity has been substantially investigated in the third-party punishment of dictator game and similar paradigm, such as ultimatum game (Boksem and De Cremer, 2010; Wu et al., 2011; Qu et al., 2013; Sun et al., 2015; Mothes et al., 2016). The FRN was extracted immediately after the receiver realized the fair or unfair offer in the ultimatum game paradigm, or the third-party actor witnessed the assignment of the dictator in the third-party punishment of dictator game. These studies concluded that unfair offers or assignments elicited more pronounced FRN amplitude compared with fair offers. However, we cannot use this idea to interpret our results because in our experimental paradigm, which was adopted from the third-party punishment of dictator game, the FRN was evoked by the third-party behaviors instead of the dictator’s offers. Moreover, when the fourth-party bystanders evaluated third-party behaviors, they faced the same assignment from the dictator. Even now, the analysis of FRN composition was still useful in our study. Because except for unfairness, previous studies also found that FRN is sensitive to negative outcomes (Boksem and De Cremer, 2010), others’ negative situations (Yeung et al., 2005; Koban and Pourtois, 2014; Wei et al., 2015), or unexpected events (Oliveira et al., 2007; Sun et al., 2015; Mothes et al., 2016). Thus, in the present study, larger FRN values reflected that TPH somewhat violated the expectancy of the fourth-party bystanders.

### Second Expectation: FRN Characteristics May Not Predict Fourth-Party Evaluation Scores

We found that the ERP result showed that third-party punishment was more likely to be expected by the fourth-party bystanders than third-party help. This result was somewhat not in accordance with the behavioral result, which showed that fourth-party bystanders agreed to third-party help more than to punishment. Accordingly, the final behavioral results showed that fourth-party bystanders agreed to third-party help more, whereas the temporary ERP responses reflected that the bystanders did not expect third-party actors to help the victims at first, which appeared to agree with our second expectation.

The case wherein FRN amplitudes did not predict final behaviors was also observed in some other studies. For example, Mothes et al. (2016) found that larger FRN amplitudes were not associated with third-party punishment. Larger FRN were elicited by unfairness, and those of third parties that would not make pro-social punishment because their levels of involvement were low. Boksem and De Cremer (2010) made another interpretation to this phenomenon; they believed that the ERP technology had high temporal resolution, which can be used to evaluate the processes immediately after the event (fair and unfair assignment). Thus, the FRN, which was locked to the witness of the dictator’s decisions, was elicited by the evaluation of the fair or unfair assignment and would not be influenced by the response preparation, which would take place at least several seconds later.

We partly followed the ideas of Boksem and De Cremer (2010) and introduced dual-process system theory to understand why third-party help elicited larger FRN, which indicated that third-party help was against with the expectation of the fourth party but acquired more agreement finally. We suspected that something happened during the process of the subconscious evaluation of third-party pro-social behaviors turning into evaluation behaviors. From a perspective of dual-process system to decision making, the evaluation decision of the fourth party was made by the interaction of two different processing systems, which were called the automatic and the controlled systems (Lieberman et al., 2007; Adolphs, 2009; Qu et al., 2014; Yang et al., 2017). Automatic system was considered to be a fast, spontaneous, short-sighted, and intuitive system, which seldom require cognitive resources. The automatic system was also called the affective system because it was mostly driven by affections or emotions (Cappelletti et al., 2011). By contrast, controlled system requires quantities of cognitive resources; thus it is deliberate, effortful, and slow. Previous studies also called the dual-process system as two-step process system (Cappelletti et al., 2011; Rand et al., 2012; Sun et al., 2015; Yang et al., 2017). Intuitive proposals were made automatically during the first step. In the second step, actors made tradeoffs between the proposals from the first step. In this deliberative step, motivational consideration, social contextual consideration, and quantities of cognitive resources were injected.

The third-party punishment and expectation of third-party punishment might be related to the automatic system or proposals generated in the first step, as they were considered to be driven by emotional factors (Qu et al., 2014). Crockett et al. (2010) believed that impulsive emotional responses induced by unfairness may play an important role in driving third-party punishment (Crockett et al., 2010). Qu et al. (2014) suggested that emotional factors were uncontrolled and automatic when participants made decisions. Emotional factors can provide great power to punish and make third-party punishment decisions spontaneously and unconsciously (Olatunji and Puncochar, 2014). On the contrary, not all punishment behaviors were cogitative and conscious. Punishment tended to be automatically taken when norm violations were witnessed. Sun et al. (2015) found that when participants yearned for fairness during an unfairness experience, a greater MFN was elicited, which was in accordance to the results of other studies (Mothes et al., 2016; Wang et al., 2016a). They also supported the idea of Qu et al. (2014) that third-party punishment, which was an automatic intuitive proposal, occurred in the early stage of the outcome evaluation. Empathic anger, which would rapidly arise upon witnessing injustice or harm to someone else, was considered a key factor for third-party punishment and third-party punishment expectation (Fehr and Gächter, 2002; Fehr and Fischbacher, 2004; Batson et al., 2007; Van Doorn et al., 2014; FeldmanHall et al., 2015). The expectation of third-party punishment was an automatic intuitive proposal and occurred in the early stage after social publics witnessed the unfair assignment decision of the first party (Qu et al., 2014; Sun et al., 2015; Mothes et al., 2016).

Different from the third-party punishment expectations, which were intuitively or subconsciously driven, the decisions and expectations of third-party help were more complex. Except for probably emotional reactions, third-party help was affected by some other factors, such as moral judgment, perceived responsibility or duty to help, or even the perceived utility of helping (Erlandsson et al., 2014). Carlo (2014) believed that pro-social help occurs in social contexts. He suggested that except for the intrinsic processes, such as sympathy, internalized values or principles, or a strong pro-social or moral identity, pro-social help may also be motivated by external or social context concerns (e.g., social approval, social power, and money). Loke et al. (2011) found that reasoning about pro-social help, which was a kind of moral judgment, played an important role in pro-social help evaluation.

Compared with the evaluations of third-party punishment, more cognitive resources were inputted by the bystanders when they evaluated third-party help decisions. Thus, the fourth-party evaluation of third-party help tended to form in the second step of decision-making or social information processes, which involved a more deliberative and controlled phase and were sensitive to social context. Therefore, immediately after witnessing the unfairness decision, fourth-party bystanders may subconsciously expect third-party actors to punish the norm violators. With more cognitive resources and moral or social context concerns related to the third-party help evaluation introduced into the evaluating process, the bystanders’ expectations or evaluations tended to change. Social public expected others to punish the norm violators immediately after witnessing a norm violation, but ultimately agreed to helpful third parties more.

### Fairness, Help, and Punishment

The present study also found that the effect of third-party behaviors on the FRN of fourth-party bystanders was modulated by first-party assignments. Based on ERP results, we found that the FRN amplitude of third-party help was significantly more negative than that of third-party punishment only when the third-party behaviors can achieve fairness between the dictator and the receiver regardless of the first-party assignment. No significant difference in the FRN amplitude was found between third-party help and punishment when third-party actors cannot safeguard fairness under condition (90:10) with help/punishment magnitude 45. We speculated that under condition (90:10) with help/punishment magnitude 45, the third-party behaviors could hardly achieve justice between norm violators and victims. As a result, bystanders tended to regard third-party help and punishment as same behaviors, and they subconsciously believed that the two behaviors were all bad choices. Only after they realized that the third-party actors can afford to safeguard fairness, they would focus on the difference between third-party help and punishment.

The present study also found that the effect of third-party behaviors on the FRN of fourth-party bystanders was modulated by first-party assignments. Based on ERP results, we found that the FRN amplitude of third-party help was significantly more negative than that of third-party punishment under condition (70:30) with help/punishment magnitude 45 and (90:10) with help/punishment magnitude 80. No significant difference in the FRN amplitude was found between third-party help and punishment under condition (90:10) with help/punishment magnitude 45. These results indicated that first-party assignments and third-party behaviors could not determine the forth-party evaluation separately. Under condition (70:30) with help/punishment magnitude 45 and (90:10) with help/punishment magnitude 80, the third-party actors could achieve fairness between the dictator and the receiver. However, third-party actors cannot safeguard fairness under condition (90:10) with help/punishment magnitude 45. Whether the third-party behaviors could achieve fairness between the dictator and the receiver is a precondition of the neural difference between third-party help evaluation and punishment evaluation.

If the third-party behaviors could hardly achieve justice between norm violators and victims, bystanders tended to regard third-party help and punishment as same behaviors and consider that the two behaviors were all bad choices. Only after they realized that the third-party actors can afford to safeguard fairness, they would focus on the difference between third-party help and punishment. The occurrence of this situation needed to be traced back to the neural processing in our brains. Kahneman (2011) suggested that our brains were tended to be lazy in our daily life to save cognition resources. Our brains preferred to encode the things we perceived into binary categories when cognition resources were limited. This phenomenon was significant in the neural processing reflected by FRN. Holroyd et al. (2006) found that FRN appeared to reflect a binary categorization of the outcomes as either good or not good. The TPH and TPP under condition (90:10) with help/punishment magnitude 45 were all “no good” behaviors for bystanders, because the third-party actors didn’t achieve justice between norm violators and victims. The forth-party bystanders would not distinguish between the two bad choices. As a result, the FRN amplitudes of TPP and TPH appeared to be the same under condition (90:10) with help/punishment magnitude 45.

## Conclusion

This study is the first to examine the neural correlations of the fourth-party evaluation of third-party pro-social behaviors using ERP technology. Behavioral results showed that fourth-party bystanders agreed to third-party help more than to third-party punishment. However, the tendency was decreased with the increase in the unfairness of the first-party assignment. Third-party help elicited more negative FRN amplitude at least under the assignment (70:30) with help/punishment magnitude of 45 and (90:10) condition with help/punishment magnitude 80. Specifically, no difference in the FRN amplitudes was observed under (90:10) with help/punishment magnitude of 45. These results indicated that, although bystanders finally agreed that third party should help the victims more, they expected third-party actors to punish the norm violators immediately after they witnessed the norm violation. However, this phenomenon appeared only when the fourth-party bystanders believed that the third-party actors can safeguard fairness.

### Limitations

Potential limitations of the studies reported here must be emphasized. In the present study, we used dual-process system theory to explain our finding that third-party help evoked larger FRN but obtained more behavioral agreement compared with punishment. We suspected that the FRN reflected a relatively automatic process during which third-party punishment was expected. However, the high evaluation score of third-party help mainly resulted from a more deliberate process. Though previous studies somewhat supported our interpretation (Boksem and De Cremer, 2010; Sun et al., 2015), direct experimental evidence that can distinguish between the proposed automatic and deliberate evaluations was still needed. Although the automatic process was difficult to orient, weakening the controlled system and the deliberative process by time pressure, cognitive load, or some other methods was possible (Cappelletti et al., 2011). Future studies can perform some of these methods to distinguish between automatic and deliberate processes and produce more persuasive results.

## Ethics Statement

This study was carried out in accordance with the recommendations of the Ethics Committee of Business School of Nankai University with written informed consent from all subjects. All subjects gave written informed consent in accordance with the Declaration of Helsinki. The protocol was approved by the Ethics Committee of Business School of Nankai University.

## Author Contributions

JL and SL conceived and designed the study. JL, SL, PW, GW, and XY designed the experimental stimuli and procedure. SL, CZ, and XL implemented experimental protocols and collected data. SL, XN, and GW analyzed the data. SL, JL, and XY wrote and revised the paper.

## Conflict of Interest Statement

The authors declare that the research was conducted in the absence of any commercial or financial relationships that could be construed as a potential conflict of interest.
